# Micro-contextual identification of archaeological lipid biomarkers using resin-impregnated sediment slabs

**DOI:** 10.1038/s41598-020-77257-x

**Published:** 2020-11-25

**Authors:** Caterina Rodríguez de Vera, Antonio V. Herrera-Herrera, Margarita Jambrina-Enríquez, Santiago Sossa-Ríos, Jesús González-Urquijo, Talia Lazuen, Marine Vanlandeghem, Claire Alix, Gilliane Monnier, Goran Pajović, Gilbert Tostevin, Carolina Mallol

**Affiliations:** 1 Archaeological Micromorphology and Biomarkers Laboratory (AMBI Lab), Instituto Universitario de Bio-Orgánica “Antonio González”, Universidad de La Laguna, Tenerife, Spain; 2 Departamento de Biología Animal, Edafología y Geología, Universidad de La Laguna, Tenerife, Spain; 3 Departamento de Historia e Historia del Arte, Universitat Rovira i Virgili, Avenida de Cataluña, 35, 43002 Tarragona, Spain; 4 Institut Català de Paleoecología Humana i Evolució Social (IPHES), Zona Educacional 4, Campus Sescelades Universitat Rovira I Virgili (Edificio W3), 43007 Tarragona, Spain; 5 Instituto Internacional de Investigaciones Prehistóricas de Cantabria, IIIPC-University of Cantabria, Edificio Interfacultativo, Universidad de Cantabria, Avenida de Los Castros, 52, 39005 Santander, Spain; 6 CNRS, MCC, PACEA, UMR 5199, Université de Bordeaux, 33600 Pessac Cedex, France; 7 UMR 7041 ArScAn, Université Paris 1 Panthéon Sorbonne, 21 allée de l’université, 92023 Nanterre Cedex, France; 8 Université Paris 1 Panthéon Sorbonne, 8096 ArchAm, 21 allée de l’université, 92023 Nanterre Cedex, France; 9 Department of Anthropology, University of Minnesota, Minneapolis, MN USA; 10 National Museum of Montenegro, Novice Cerovića, 7, 81250 Cetinje, Montenegro; 11 Departamento de Geografía e Historia, UDI Prehistoria, Arqueología e Historia Antigua, Facultad de Geografía e Historia, Universidad de La Laguna, Tenerife, Spain

**Keywords:** Biogeochemistry, Environmental social sciences

## Abstract

Characterizing organic matter preserved in archaeological sediment is crucial to behavioral and paleoenvironmental investigations. This task becomes particularly challenging when considering microstratigraphic complexity. Most of the current analytical methods rely on loose sediment samples lacking spatial and temporal resolution at a microstratigraphic scale, adding uncertainty to the results. Here, we explore the potential of targeted molecular and isotopic biomarker analysis on polyester resin-impregnated sediment slabs from archaeological micromorphology, a technique that provides microstratigraphic control. We performed gas chromatography–mass spectrometry (GC–MS) and gas chromatography–isotope ratio mass spectromety (GC–IRMS) analyses on a set of samples including drill dust from resin-impregnated experimental and archaeological samples, loose samples from the same locations and resin control samples to assess the degree of interference of polyester resin in the GC–MS and Carbon-IRMS signals of different lipid fractions (n-alkanes, aromatics, n-ketones, alcohols, fatty acids and other high polarity lipids). The results show that biomarkers within the n-alkane, aromatic, n-ketone, and alcohol fractions can be identified. Further work is needed to expand the range of identifiable lipid biomarkers. This study represents the first micro-contextual approach to archaeological lipid biomarkers and contributes to the advance of archaeological science by adding a new method to obtain behavioral or paleoenvironmental proxies.

## Introduction

In recent decades, Archaeology has been geared to a search and characterization of organic matter using different techniques. Notable progress has been made since the early stages of lipid residue analysis in the late 1960s to 80s^1–3^, and today we are able to identify proteins from archaeological bone^4–6^, as well as ancient DNA from archaeological sediment^7^. However, these applications have not taken microstratigraphic complexity into consideration. A few millimeters or centimeters of stratified archaeological sediment may represent decades, centuries, or millennia. Thus, behavioral or paleoenvironmental inference from archaeological samples requires microstratigraphic control^8^.

One microstratigraphic technique that can provide contextualized, high-temporal resolution data is archaeological soil micromorphology^9,10^. The technique relies on principles of petrography, micropedology and experimental research to identify microscopic particles and interpret geogenic, biogenic and anthropogenic processes from their spatial distribution and diagnostic porosities and microstructures. This is achieved through microscopic observation of 30 µm-thick archaeological sediment thin sections made from intact, oriented blocks of sediment collected from stratigraphic profiles or excavation surfaces. The thin sections are described using a combination of standard guidelines inherited from soil science^11^ and developed by geoarchaeologists^9,10,12^. Although micromorphology has been used by geoscientists since the early twentieth century, there is a clear trend towards its application in archaeology and in recent years, a growing emphasis on coupling micromorphological, contextualized data with microstratigraphic geochemical and geophysical data such as those obtained through X-ray diffraction and fluorescence (XRD and XRF), Raman and infrared spectroscopy (FTIR), magnetic susceptibility or gas chromatography^8,13^. Some techniques, such as µXRF, µRaman or µFTIR, allow direct analysis on thin sections and resin-impregnated micromorphology slabs, which are spectral images of thin sections^14–16^.

Archaeological soil micromorphology has shown to be a powerful tool for archaeological research. However, as a technique rooted in petrography, its strength lies in mineral identification. Visual characterization of organic particles in archaeological sediment thin sections is a challenging task, as these are isotropic under cross polarized transmitted light, and often amorphous. In addition, the embedding medium of the samples is composed of complex organic polymers^17^ such as epoxy or polyester resins, which have possibly deterred microanalytical investigations of archaeological organic matter on micromorphological samples. As a result, little advance in incorporating other microanalytical techniques to the micro-contextual, micromorphological approach has been made.

Combining data from archaeological soil micromorphology and gas chromatography holds great potential for archaeological interpretation^18–20^. Micromorphology provides high resolution contextual data and gas chromatography provides molecular information on lipid content, particularly if coupled with isotopic ratio mass spectrometry (IRMS) for compound specific isotope analysis (CSIA)^21–23^. Lipids are organic molecules that preserve well in sedimentary contexts for long periods of time^24–26^ and they can be informative of human activities, diets and paleoenvironments^27–29^. However, coupling chromatography with micromorphology in a similar way as has been achieved with other techniques (e.g., µFTIR, µRaman or µXRF) is challenging due to the presence of resin in micromorphological samples. So far, only side-by-side sampling with micromorphology blocks and adjacent loose archaeological sediment for lipid analysis has been possible. Although side-by-side sampling has produced useful archaeological and paleoenvironmental information^19,30–32^, the results have unknown degrees of micro-contextual inaccuracy.

Although organic resins are an important source of interference in the characterization of lipids contained in micromorphological samples, their use as consolidating agents is still preferable to silica-based resins, which have low penetrability and an higher cost^33^. An organic resin is, basically, a mixture of low molecular weight molecules (monomers) that can bond to others creating a solid polymer^34^. Phthalates and other plasticizers are included in the mixture to enhance properties such as low degradation, flexibility, extensibility and workability^35,36^. Other additives such as triphenyl stibine or hydrocinnamic acid are also added as flame retardants or protectors. The catalyst used in the mixture could also contain phlegmatizers such as alkylene glycols and esters (mainly phthalates)^37^. All these compounds have similar polarity and chromatographic retention times as lipid molecules. However, the extent to which the similarities might represent an obstacle in lipid biomarker identification has not been explored. Determining the interference effect of polyester resin on GC–MS and IRMS signals is key to achieve direct lipid analysis on micromorphological resin-impregnated slabs.

In this paper, we present results of experiments designed to assess the degree of interference of polyester resin in the GC–MS and Carbon-IRMS signals of different lipid fractions: n-alkanes (F1), aromatics (F2), n-ketones (F3), alcohols (F4), fatty acids (F5) and other high polarity lipids (F6). Our goal is to assess the possibility of identifying archaeological lipid compounds directly from resin-impregnated micromorphology slabs produced in thin section manufacture.

## Results

The compounds identified in this study are listed in supplementary documents Sup-2 (polyester resin compounds) and Sup-3 (archaeological lipid biomarkers). To facilitate the identification of archaeological lipid biomarkers, Extracted Ion Current (EIC) chromatograms were obtained attending to the key ions for each lipid type. In the following paragraphs, we summarize the main findings regarding resin constituents, biomarkers identified within the lipid fractions, and the fatty acid carbon isotopic values obtained.

### Identification of resin constituents

We identified the main polyester resin constituents, plasticizers and byproducts in the lipid fractions of resin-containing samples: (1) control samples and (2) experimental and archaeological drill dust (DD) samples (see Sup-2). Benzoic acid, phthalic anhydride, phthalic acid, dimethyl phthalate and dioctyl phthalate were identified as the predominant resin constituents. Plasticizers including methyl-2-ethylhexyl phthalate, diallyl phthalate, 2,2,4-trimethyl-1,3-pentanediol diisobutyrate, hexanedioic acid, bis(2-ethylhexyl) ester, monoisopropyl phthalate, allyl methyl phthalate, monoethyl phthalate and methyl phthalyl ethyl glycolate were also found. Benzophenone-3, 2-(2′-hydroxy-3′,5′-di-tert-amylphenyl) benzotriazole, chalcone and cinnamic acid were identified as compounds with UV stabilizer properties. Dicarboxylic acids including fumaric, isophthalic and terephthalic were also identified. We also found other substances commonly present in unsaturated resins such as α N-normethadol, thiocarbamic acid, n,n-dimethyl, S-1,3-diphenyl-2-butenyl ester and Benzene, 1,1′,1′-[5-methyl-1-pentene-1,3,5-triyl]tris. The highest peaks in our chromatograms seemed to be mixtures of different overlapping phthalates.

### n-Alkane characterization

In F1, we only identified one untargeted compound: diisooctyl phthalate in R1. In this sample, n-alkanes (EIC *m/z* 43, 57, 71, 85) ranging between C_22_ and C_33 in_ were identified. Salt-1 yielded n-alkanes between C_18_ and C_33_ in the loose sediment (LS) sample and only from C_20_ to C_29_ in the corresponding DD. For CS LS samples, we identified n-alkanes from C_19_ to C_33_, from C_21_ to C_31_ in the AX1 LS sample and from C_21_ to C_33_ in AX2 and AX3 LS samples. In all these cases, the same n-alkanes were found in their corresponding DD samples (see Sup-3). The highest peaks registered in the LS and DD chromatograms of the AX2 and AX3 samples, corresponding to C_29_ and C_31_ n-alkanes.

No n-alkanes signals were detected in LS or DD from samples SMS, Salt-2 and SC. They were also not detected in control samples R2, R3 and R4.

### Aromatic hydrocarbon, n-ketone and alcohol identification

Biomarkers are listed in Sup-3. In F2, Pyrene was the only aromatic compound detected in this study (SC DD sample). This compound is a combustion biomarker or pyromarker included in the polycyclic aromatic hydrocarbon group (PAHs)^38^.

In F3, two long-chain n-ketones, 16-hentriacontanone and 18-pentatriacontene, were detected in the SMS sample with the EIC measure (*m/z* 239, 255, 268, 283).

F4 showed, together with F5, the most complex chromatograms. In F4, we studied the TICs (Total Ion Current) chromatograms and EICs separately. The chromatograms of the LS samples allowed us to spot the presence of linear alcohols (n-alkanols) with even numbered carbon predominance in five of the samples. Despite the observed prevalence of resin-related compounds, the DD homologues samples also yielded several biomarkers after EICs from selected key ions of previously identified n-alkanols (nC_16_-OH to nC_30_-OH; *m/z* 75, 299, 327, 355, 383, 411, 439, 467, 495). These biomarkers are detailed below.

In the Salt-1 LS sample, we observed nC_14_-OH, nC_16_-OH and nC_18_-OH, whilst nothing was found in its corresponding DD. In the CS LS sample, we identified even-numbered carbon chain n-alkanols ranging from nC_16_-OH to nC_30_-OH with highest peaks in nC_26_-OH and nC_30_-OH. EIC analysis of the CS DD sample, reveals the presence of low intensity peaks in the same retention time (RT) for nC_20_-OH, nC_26_-OH, nC_28_-OH and nC_30_-OH as those recorded in the CS LS sample. For AX1, AX2 and AX3, the LS samples revealed the presence of even-numbered linear alcohols ranging from nC_22_-OH to nC_30_-OH, with the highest peak in nC_26_-OH. The predominant n-alkanol (nC_26_-OH) was also found in the DD homologue sample. Finally, we observed nC_14_-OH, nC_16_-OH and nC_18_-OH in the SMS sample. No n-alkanol was found in samples related to Salt-2 (LS and DD).

In the F4 from CS LS, traces of two monoacylglycerols also known as MAGs (glycerol monoestearate and glycerol monopalmitate) were also found in the TIC chromatogram, whilst no signs of these compounds were observed in CS DD. In Axlor LS samples, glycerol monoestearate and glycerol monopalmitate were found along with glycerol and β-sitosterol. In their DD homologues, we identified the same MAGs as in LS samples and glycerol but no β-sitosterol.

In SC samples (LS and DD) no linear alcohols signal were observed and only cholesterol was identified in the DD sample.

### Fatty acid characterization

F5 and F6 yielded similar results (see Sup-3), and the presence of resin constituents was higher in F5 than in F6. Applying EICs for key ions *m/z* 74, 87, 101 allowed us to identify C_16:0_ and C_18:0_ FAMEs in all the resin control samples. Additionally, an α-ω-dicarboxylic acid, nonanedioic acid (azelaic acid) which is a common archaeological lipid biomarker^21^ was found in R1 and R4.

Examining the EIC performed on the remaining samples, C_16:0_ and C_18:0_ FAMEs were present in all, the LS and the DD samples. In Salt-1, we also identified the correspondent derivatized form of the triterpenoid oleanolic acid. C_14:0_, C_16:0_ and C_18:0_ FAMEs were found in the CS LS and DD samples. The SMS sample also yielded C_14:0_, C_16:0_ and C_18:0_ FAMEs, along with C_20:0_, C_22:0_ and C_24:0_ and other oxidized FAMEs such as 10-hydroxy-hexadecanoic acid and 10-hydroxy-octadecanoic acid. In SC, several free fatty acid FAMEs were identified including, C_14:0_, C_16:0_, C_16:1Δ9_, C_18:0_, C_18:1Δ9_, C_18:1Δ11_, C_18:2Δ9,11_ and C_20:0_, in both, the LS and DD samples. C_20:4Δ5,8,11,14_ FAMEs and two α-ω-dicarboxylic acids, azelaic acid and undecanedioic acid, were also found in the SC LS sample. In the SC DD sample, C_15:0_, C_17:0_, C_18:2Δ9,12_, C_20:1Δ11_, C_22:0_ FAMEs, decanedioic acid (sebacid acid) and undecanedioic acid were also present.

### δ^13^C_16:0_ and δ^13^C_18:0_ values

δ^13^C_16:0_ and δ^13^C_18:0_ values were obtained for all the resin control samples (R1 to R4) and for CS, AX1, AX2, AX3 and SMS LS and DD samples (Table 1). In Salt-1 and Salt-2 samples (LS and DD), concentrations of C_16:0_ and C_18:0_ were insufficient to perform the IRMS analysis. The resin control and DD samples were corrected for the Suess effect and the corrected δ^13^C values are presented in Table 2.

**Table 1:** C_16:0_ and C_18:0_ fatty acid δ^13^C values of calculated for LS and DD samples without Suess effect correction.

	Loose sediment (LS)					Drill dust (DD)				
	δ^13^C_16:0_ (‰) VPDB	σ	δ^13^C_18:0_ (‰) VPDB	σ	Δ^13^C (δ^13^C_18:0_ ‰ − δ^13^C_16:0_ ‰)	δ^13^C_16:0_ (‰) VPDB	σ	δ^13^C_18:0_ (‰) VPDB	σ	Δ^13^C (δ^13^C_18:0_ ‰ − δ^13^C_16:0_ ‰)
**Without Suess effect correction**										
AX1	− 32.9	0.5	− 32.9	0.2	0.0	− 33.1	0.2	− 31.0	0.5	2.1
AX2	− 33.2	0.3	− 33.3	0.0	− 0.1	− 34.3	0.4	− 29.9	0.2	4.4
AX3	− 32.2	0.3	− 32.9	0.5	− 0.8	− 33.8	0.5	− 30.5	0.1	3.3
CS	− 30.9	0.4	− 31.8	0.5	− 0.9	− 32.2	0.1	− 31.3	0.5	0.9
SMS	–	–	–	–	–	− 29.2	–	− 30.4	–	− 1.3
R1	–	–	–	–	–	− 34.6	0.1	− 32.0	0.5	2.5
R2	–	–	–	–	–	− 30.5	0.2	− 31.1	0.2	− 0.6
R3	–	–	–	–	–	− 29.2	0.4	− 31.2	0.3	− 1.4
R4	–	–	–	–	–	− 31.3	0.2	− 31.8	0.4	− 0.5

**Table 2:** C_16:0_ and C_18:0_ fatty acid δ^13^C values calculated for BS and DD samples with Suess effect correction*.*

	Loose sediment (LS)					Drill dust (DD)				
	δ^13^C_16:0_ (‰) VPDB	σ	δ^13^C_18:0_ (‰) VPDB	σ	Δ^13^C (δ^13^C_18:0_ ‰ − δ^13^C_16:0_ ‰)	δ^13^C_16:0_ (‰) VPDB	σ	δ^13^C_18:0_ (‰) VPDB	σ	Δ^13^C (δ^13^C_18:0_ ‰ − δ^13^C_16:0_ ‰)
**With Suess effect correction**										
AX1	− 32.9	0.5	− 32.9	0.2	0.0	− 31.2	0.2	− 29.1	0.5	2.1
AX2	− 33.2	0.3	− 33.3	0.0	− 0.1	− 32.4	0.4	− 28.0	0.2	4.4
AX3	− 32.2	0.3	− 32.9	0.5	− 0.8	− 31.9	0.5	− 28.6	0.1	3.3
CS	− 30.9	0.4	− 31.8	0.5	− 0.9	− 30.3	0.1	− 29.4	0.5	0.9
SMS	–	–	–	–	–	− 27.3	–	− 30.9	–	− 1.3
R1	–	–	–	–	–	− 32.7	0.1	− 30.1	0.5	2.5
R2	–	–	–	–	–	− 28.6	0.2	− 29.2	0.2	− 0.6
R3	–	–	–	–	–	− 27.9	0.4	− 29.3	0.3	− 1.4
R4	–	–	–	–	–	− 29.4	0.2	− 29.9	0.4	− 0.5

The δ^13^C values of LS from archaeological samples ranges between − 30.9 ± 0.4 and − 33.2 ± 0.3‰ for δ^13^C_16:0_ and between − 31.8 ± 0.5 and − 33.3 ± 0.0 ‰ for δ^13^C_18:0_. For the corresponding DD samples, after Suess effect correction, the values oscillate between − 30.3 ± 0.1 and − 32.4 ± 0.4‰ for δ^13^C_16:0_ and − 28.6 ± 0.1 and − 29.4 ± 0.5‰ for δ^13^C_18:0_. In the resin control samples, δ^13^C_16:0_ values range from − 27.9 ± 0.4 to − 32.7 ± 0.1 and from − 29.2 ± 0.2 to − 30.1 ± 0.5 for δ^13^C_18:0_ after the correction.

### Statistical analyses

Statistical analyses were performed to estimate if the n-alkanes present in a control sample could interfere with the archaeological DD n-alkane profile and to assess the significance of the differences between LS and DD δ^13^C values.

First, we analyzed the normalized areas (A_n-alkane_/A_IS_) of the target compounds by comparing R1 to Axlor and Crvena Stijena DD samples. n-Alkanes ranging from C_22_ to C_33_ were considered in all the samples except for AX1 DD, in which the analysis was carried out on n-alkanes from C_22_ to C_31_. The results showed that the n-alkane distribution is significantly different between R1 and AX2 DD and CS DD samples, but no significant differences were obtained when comparing R1, AX1 DD and AX3 DD (Table 3).

**Table 3:** Results of a non-parametric test applied to n-alkane distributions. H_0_ is assumed when p-value > α.

Sample	Bilateral test (α = 0.05)				Wilconxon test (α = 0.05)			
	N+	Expected value	Var (N+)	p-value (bilateral)	V	Expected value	Var (V)	p-value (bilateral)
CS	0	6.00	3.00	0.00	0	39.00	162.50	0.00
AX1	3	5.00	2.50	0.34	24	27.50	96.25	0.77
AX2	1	6.00	3.00	0.00	6	39.00	162.50	0.00
AX3	3	6.00	3.00	0.14	16	39.00	162.50	0.07

In the second analysis, the results indicate that there is no significant difference between LS and DD δ^13^C_16:0_ values (p value < α) with or without a Suess effect correction. However, for C_18:0_ the differences between LS and DD samples are significant independently of the Suess effect correction (p value > α).

## Discussion

Characterizing archaeological biomarkers directly obtained from sediment resin-consolidated slabs is possible, although certain aspects should be considered. Here, we discuss first the effect of the solvents used, followed by the archaeological biomarkers found in the different lipid fractions and finally the CSIA results and statistics.

### Effect of the solvents used in this study

Hexane is the least polar of the solvents employed in our study (Table 4) and has a low potential to dissolve polyester resins^39^. As a result, fractions that eluted with high percentages of n-hexane (F1 and F2) showed less presence of polyester resin related compounds (see Sup-2). Dichloromethane (DCM) which was also used in this study, is a volatile solvent that can promote changes in the resin molecular microstructure^34^. During extraction of the resin control samples and their elution into the silica chromatography columns, we observed changes in the aggregation of the drill dust, indicating the occurrence of molecular changes and their possible effect on the Total Lipid Extract (TLE) and in the different fractions eluted with high percentages of DCM. On the other hand, experimental and archaeological DD samples, which are a mix of the resin constituents and sedimentary compounds, yielded a weaker solvent effect when processed. Thus, we could identify a substantial concentration of resin constituents in some fractions, mainly from F4 onwards. As resin constituents are molecules with similarities to our target lipids, we encountered interference when trying to identify certain biomarkers particularly n-alkanols and fatty acids, which co-eluted with phthalates and/or other resin constituents. In this case, analyzing the EIC allowed us to analyze the samples further.

**Table 4:** Lipid fraction chromatography.

Fraction number	Lipid family by functional groups	Elution volume (mL)	Mobile phase
1	n-alkanes	1.7	n-hexane
2	Aromatics	3.4	n-hexane: DCM (8/2, v/v)
3	Ketones	3.4	DCM
4	Alcohols	3.4	DCM: Ethyl acetate (EtOAc) (1/1, v/v)
5	Fatty acids	3.4	EtOAc
6	Other lipids with higher polarity	3.4	DCM: MeOH (7/3, v/v)

### n-Alkanes

**Figure 1:**
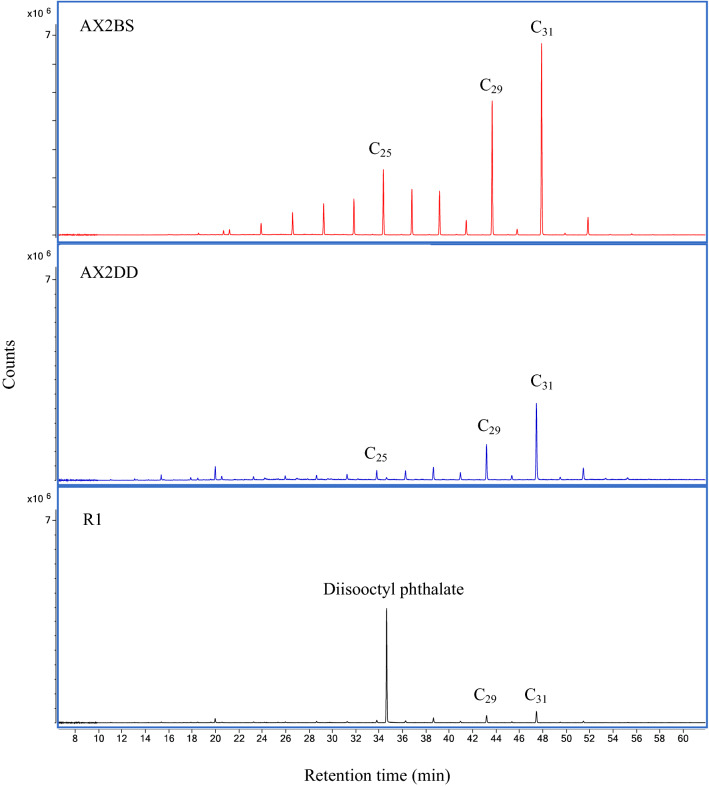
Extracted Ion Chromatograms from samples AX2 BS, AX2 DD and R1. In the n-alkane fraction (F1) of R1 only one phthalate was characterized along with some n-alkanes. The AX2 LS and DD sample chromatograms share similarities among their n-alkanes profiles.

Of all the F1 samples examined, only R1 showed a phthalate (diisoctyl phthalate), and it also contained traces of n-alkanes. Interestingly, despite the presence of this type of compound in one resin control sample, it was possible to discern archaeological n-alkanes in the DD samples based on the differences between the resin control sample and the corresponding DD and LS samples, as can be seen in Fig. 1. This figure presents a comparison of F1 chromatograms from the AX2 samples (LS and DD) and shows their similarity. Dominant n-alkanes C_29_ and C_31_ peaks suggest presence of vascular land plant biomarkers^3,40^. As R4 did not yield any n-alkanes, we compared AX2 samples with n-alkanes present in the control sample R1. As can be seen in Fig. 1, although the dominant n-alkanes are the same, the distribution and intensity of the peaks are different. These results, altogether with the statistical analysis, suggest the need for statistical data treatment in subsequent interpretations.

### Aromatic hydrocarbons

Pyrene, which was the only aromatic molecule found, is a PAH^38^. These compounds are present in natural resources such as petroleum, but they are also produced during incomplete combustion processes^41^. Pyrene has been characterized on virgin polystyrene (PS) and other plastics can also act as sink for certain pyrene-related compounds^42^. However, we did not find pyrene or other PAHs in any of our resin control samples. It was found in the SC DD sample, in agreement with the charred nature of the sample. Furthermore, pyrene is one of the most abundant PAHs in coastal zones and its presence in fish has been previously documented^38^. Absence of pyrene in the unconsolidated SC sample could be explained by the volatile nature of this compound, which would result in an irregular distribution^43^.

### n-Ketones

Two long-chain n-ketones, 16-hentriacontanone and 18-pentatriacontanone, were identified in the SMS sample. Their formation is related with thermal alteration, resulting from ketonic decarboxylation of fatty acids^44^. They are biomarkers of vegetable cooking practices^21^ and also have been reported in experimental fireplaces fueled with fresh bone^45^. Long-chain n-ketones were not detected in our resin control samples and we did not find any literature about their use in polyester resins. Thus, their presence in our SMS can be interpreted as archaeological. Further micro-contextual investigation is needed to test this possibility.

### Alcohols

As in the previous fraction, n-alkanols were not detected in any of our resin control samples and there is no evidence in the literature about their presence in polyester resins. Long-chain linear alcohols (> nC_20_-OH) with a strong predominance of even carbon-numbered chains are considered to be terrigenous input biomarkers because their presence is linked to the existence of vascular plant waxes, while their shorter homologues are related to microbial degradation, phytolipidic metabolism and animal organic matter degradation^46–48^. Our Axlor LS samples yielded long-chain linear alcohols with predominant nC_26_-OH, pointing to vascular land plant waxes as their possible origin. In the corresponding DD samples, despite the low concentration, we observed at least the predominant n-alkanol (nC_26_-OH) in AX2 and AX3. The CS LS sample yielded a wide range of n-alkanols indicating not only the existence of vascular plant waxes, but also degradation processes. In the DD chromatogram, several of those n-alkanols were also detected. In both cases, homologues samples suggested the same origin. The source of the n-alkanols identified in SMS sample (nC_14_-OH, nC_16_-OH and nC_18_-OH) is related to the thermal degradation of animal organic matter. Finally, the absence of n-alkanols in SC samples can be attributed to their low concentration in the sample. We recommend to study F4 with caution, as these particular biomarkers overlap strongly with phthalates (they share both the RT and key ion *m/z* 75).

Regarding the presence of MAGs in the Axlor (LS and DD) and Crvena Stijena (LS and DD) samples, they are indicators of triacylglycerol degradation^21,49^. Here too, presence of glycerol and glycerol monostearate should be taken with caution, as they are sometimes added in certain organic resins, mainly saturated resins^50,51,56^. As the organic resins used for micromorphological thin section manufacture are usually unsaturated, they should not contain MAGs. Thus, their presence in Axlor and Crvena Stijena DD samples can be interpreted as archaeological biomarkers. In-depth analysis of the corresponding geoarchaeological context is needed to explore this possibility.

The concentration of biomarkers in the samples should be considered. In our study, F4 biomarkers in LS samples showed medium–low intensity peaks (sample weight 5 g). Thus, absence or very low intensity peaks in the corresponding DD samples (sample weight 0.2 g) is expected. Finally, sterols and stanols, which are important archaeological biomarkers^52,53^, were only found in two samples: cholesterol in SC DD and β-sitosterol in AX2 LS. This study should be expanded to include samples enriched in sterols and stanols and explore their resulting signals.

### Fatty acids

Unsaturated fatty acids n-hexadecanoic acid (C_16:0_) and n-octadecanoic acid (C_18:0_), which are ubiquitous in nature, are commonly dominant in archaeological deposits and usually indicate degradation of more complex lipids^22,45,54^. We identified them in all the samples, as well as in our resin control samples. Other fatty acids such as C_12:0_, C_18:0_, C_18:1_, C_18:2_ are sometimes used in unsaturated polyester resin preparation, although they were not found in our resin control samples^34,55^.

Our SMS, CS DD and SC DD samples also yielded C_14:0_, which is another product of the triacylglyceride degradation^21^ and was not identified in the resin control samples. Other long-chain fatty acids identified in our SC (LS and DD) and SMS samples, including C_20:0_, C_22:0_ and C_24:0_ were also not detected in the resin control samples, indicating their reliability as archaeological biomarkers. SC, which is an experimental charred fish sample, yielded several saturated and polyunsaturated fatty acids, which are common lipids in salmon^56,57^, even in the DD sample. These long-chain fatty acids are indicators of an animal-derived source and have been reported in archaeological pottery residue analysis, indicating their preservation potential under certain conditions^58–61^. This agrees with our results, as the SMS resin-impregnated slab contained visible amounts of burned bone, is associated with a cold sedimentary environment (NW Alaska) and is relatively recent, between 1255 and 1330 A.D.^62^.

The experimental SC LS and DD samples also yielded presence of α-ω-dicarboxylic acids, specifically nonanedioic acid (azelaic acid), decanedioic acid (sebacic acid) and undecadioic acid. Such compounds are naturally present in plant oils or may form from heating of animal fats^23,51,59,61^, which is our case. Azelaic and sebacic acid are sometimes used in organic polymeric resin mixtures^63^, and specifically azelaic acid was identified among the resin control samples (R1 and R4), hindering the possibility of using α-ω-dicarboxylic acids as potential biomarkers in DD samples.

Oleanolic acid, found exclusively in the Salt-1 DD sample, is a triterpenoid often found among epicuticular waxes of a wide variety of plants^64^. It was not found in any of the resin control samples. Thus, it can be interpreted as a terrestrial plant biomarker. Although it was not found in the homologue LS sample, the presence of triterpenoids in sediments from El Salt site have been previously reported^19^. A possible explanation for the absence of oleanolic acid in Salt-1 LS sample is inaccurate microstratigraphic correlation between the DD and LS samples.

### Compound-specific isotopic values

Our results show that consolidating micromorphology blocks with organic polyester resin influences the C_16:0_ and C_18:0_ isotopic values (Tables 1, 2). Both fatty acids are used in resin preparation as diluents; as a way to reduce the proportion of styrene, which is classified as a hazardous air pollutant and volatile organic compound. As previously mentioned, the main sources of those fatty acids in resins are plant oils^55^.

**Figure 2:**
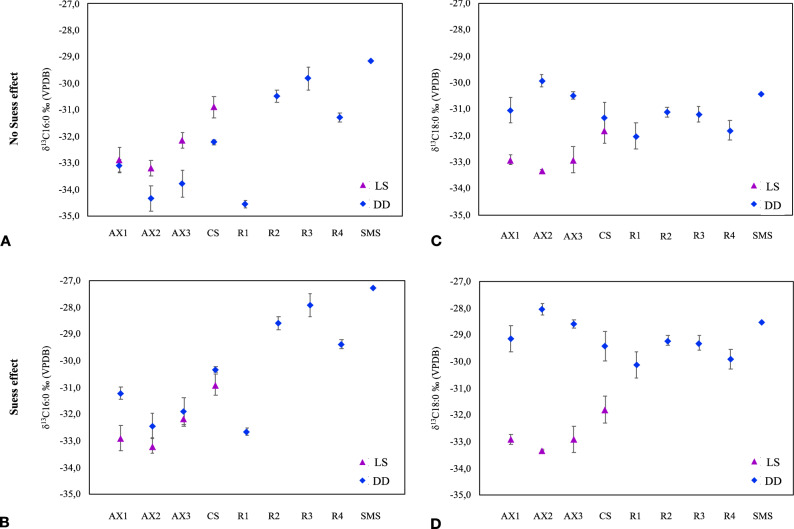
δ^13^C_16:0_ and δ^13^C_18:0_ values from Axlor, Crvena Stijena LS and DD samples, Cape Espenberg DD and resin control samples. (**A**) δ^13^C_16:0_ before Suess effect correction; (**B**) δ^13^C_18:0_ before Suess effect correction; (**C**) δ^13^C_16:0_ after Suess effect correction and (**D**) δ^13^C_18:0_ after Suess effect correction.

For δ^13^C_16:0_, values registered by DD samples are more negative than those registered for LS samples when the Suess effect is omitted. When the correction is applied, a change is observed, and the isotopic values are slightly less negative for the DD samples compared to the LS samples (differences between DD and LS δ^13^C_16:0_ isotopic values ranged from 1.32 to 1.46‰ without correction and from − 0.9 to − 0.6‰ with correction). This correction seems to particularly affect the AX1 DD sample, which showed the biggest difference after performing the Suess effect correction (Fig. 2a,c) in comparison with its LS homologue sample. In the case of δ^13^C_18:0_, values are less negative than their corresponding LS samples independently of the application of Suess Effect correction but the differences between the values of DD an LS samples increases if the correction is made (differences between DD and LS δ^13^C_18:0_ isotopic values vary between − 0.49 and − 2.25‰ without the correction and between − 2.4 and − 4.4‰ with the correction). These results indicate that, although the use of organic resin influences both isotopic signals, it affects mainly the δ^13^C_18:0_ isotopic values. The plots in Fig. 2 illustrate this point: δ^13^C_18:0_ isotopic values are closer to the resin control values whilst δ^13^C_16:0_ isotopic values are closer to the LS sample values. Regarding the Suess effect correction, as shown in Fig. 2, applying the correction is necessary to avoid incorrect interpretations in terms of ^13^C enrichment, especially for C_16:0_. To confirm the resin effect in compound-specific isotopic values in the samples analyzed by IRMS, δ^13^C_16:0_ and δ^13^C_18:0_ values of all the samples were tested statistically (Table 5). The statistical data supporting our previous observations that the resin influences the C_18:0_ isotopic signal.

**Table 5:** Results of a non-parametric test (Mann Whitney, α = 0.05), applied to δ^13^C values on AX1, AX2, AX3 and CS DD and LS samples. H_0_ is assumed when p-value > α.

	U	U (standardized)	Expected value	Var (U)	p-value
**Suess effect no considered**					
C_16:0_	13	0.00	8.00	12.00	0.20
C_18:0_	0	0.00	10.00	16.66	0.01
**Suess effect considered**					
C_16:0_	4	0.00	8.00	12.00	0.34
C_18:0_	0	0.00	8.00	12.00	0.03

**Figure 3:**
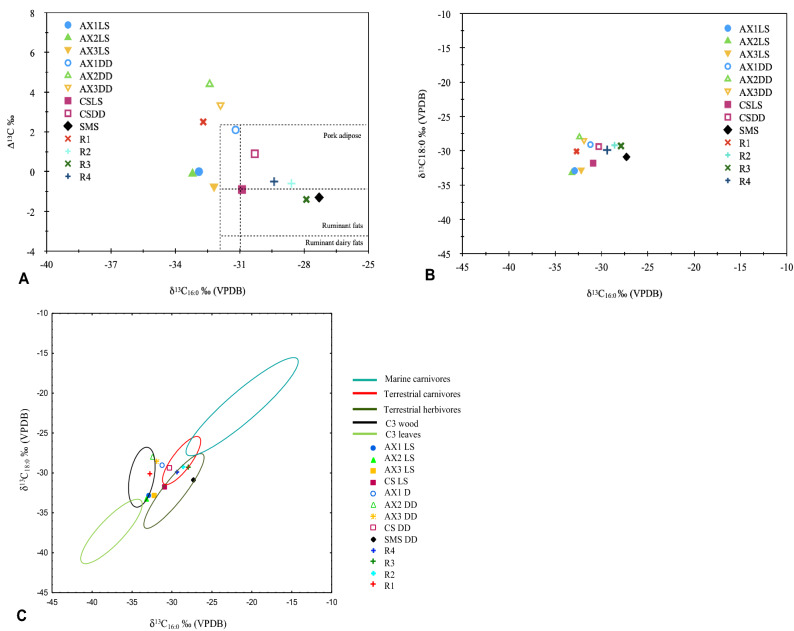
Scatter plots of ∆^13^C *vs* δ^13^C_16:0_ (**A**) using modern animal samples as reference^87,88^ and δ^13^C_16:0_/δ^13^C_18:0_ ratios (**B**) and comparison with 95% confidence ellipses plotted using published data on isotopic ratios for C3 leaves and wood, terrestrial herbivores and carnivores and marine carnivores (**C**)^21,22,65,67,69–71,89^.

Using different plots commonly used in archaeological lipid analysis^21–23,65^ also show that the differences in the position of our LS and DD samples (Fig. 3a–c) are mainly caused by the effect of the resin in the C_18:0_ isotopic signal. In Fig. 3a, positions of DD samples are different from their LS homologues samples, mainly in the Y-axis (Δ^13^C = δ^13^C_16:0_ − δ^13^C_18:0_) recording higher values. Significant differences in Δ^13^C values hamper our interpretation of possible fatty acid sources (non-ruminant domain, ruminant domain and ruminant dairy fats domain). Interestingly, control samples R2, R3 and R4 plot near one other and R1 is an outlier, emphasizing the need to include resin control samples as a part of the study. A similar pattern in the distribution of our DD and LS samples can be observed in Fig. 3b, which compares δ^13^C_16:0_ vs δ^13^C_18:0_. The bias produced by the δ^13^C_18:0_ is also noticeable in this plot.

In Fig. 3c we included confidence ellipses that may help in identify possible sources of C_16:0_ and C_18:0_ fatty acids. The figure shows that the fatty acids from resin control samples (R2 to R4) plot in the upper region of terrestrial herbivores^21,22,65–71^, whilst R1 plots in the C3 wood ellipse^22^. The C_16:0_ and C_18:0_ values of certain seeds also plot in this region^22,65,66,68,72^, confirming the plant origin of the fatty acids in the resin. Regarding the differences between our DD and LS samples, all the paired samples plot in the same ellipse, including AX2 DD and LS samples, or at least in the surrounding zones, as the AX3 (DD and LS) and CS (DD and LS) samples. More controversial is the position of AX1-related samples. As previously discussed, the variation could be a result of applying the Suess effect correction. Possibly, in AX1 DD, the proportion of archaeological FAMEs is higher than the proportion of FAMEs from organic resin, reducing the differences between DD and LS samples. Finally, SMS plots in the terrestrial herbivore domain.

Despite the biases in δ^13^C_18:0_, previous studies suggest that δ^13^C_16:0_ values can be used to discern between plant and animal sources in organic residues^22^. Our SMS sample, with a δ^13^C_16:0_ of − 29.2‰ (< − 31‰) points to presence of animal fat.

## Conclusions

In this study, we have shown the viability of lipid biomarker analysis on drill dust from resin-impregnated archaeological sediment slabs associated to micromorphological thin sections. n-Alkane characterization was successful after statistical comparison with resin control samples. We also identified aromatic compounds in the same way. Long-chain n-ketones, n-alkanols, long-chain fatty acids and triterpenoids yielded very good results, as the source of these compounds is unrelated to the resin. n-Alkanols and fatty acids eluted under the same conditions as the majority of organic resin constituents. Therefore, EIC chromatograms were required for their identification. Other biomarkers identified in this study are MAGs and α-ω-dicarboxylic acids, which are common organic resin additives and thus were checked against resin control samples. Finally, fatty acid isotopic δ^13^C values showed to be altered by the resin, also affecting the different ratios used to discriminate between lipid sources^60^.

Further research is needed to expand the list of biomarkers characterized with this approach (eg. steroids) and to obtain a mathematical model that corrects the influence of the organic resin on the n-alkane profile and on the C_16:0_ and C_18:0_ isotopic δ^13^C values. Regarding the implications of our archaeological findings, they are preliminary and require further multi-proxy research. Ongoing micro-contextual investigations of the different sites will narrow down the possible sources of organic matter revealed by this study and provide behavioral and paleoenvironmental meaning to the results. Our data add to a growing number of proxies to investigate past human behavior and paleoenvironments from a micro-contextual approach, contributing to the methodological advance of archaeological science.

## Materials and methods

### Sample selection

Table 6 presents a list of the samples studied, which includes:

1. Seven samples of polyester resin-impregnated archaeological sediment slabs corresponding to micromorphological thin sections with distinct organic-rich microfacies.
2. Six samples of loose sediment (LS) collected from the same facies and location as the micromorphology blocks associated with the selected resin-impregnated sediment slabs. One instance (sample SMS) did not have associated loose sediment.
3. A piece of experimentally produced salmon meat char.
4. The same piece of salmon meat char embedded in polyester resin.
5. Four hardened resin control samples. Three of these were from the same resin-impregnated archaeological sediment slabs.

**Table 6:** List of samples analyzed in this study.

Sample ID	Type	Description
R1	DD	Hardened resin
R2	DD	Hardened resin from a corner of a resin-impregnated archaeological sediment slab. Crvena Stijena Middle Palaeolithic site, Western Montenegro, thin section CS-MM2-B4
R3	DD	Hardened resin from a corner of a resin-impregnated archaeological sediment slab. Cape Espenberg, Northwestern Alaska (from 12 to 14th A.D.), thin section SMS-1
R4	DD	Hardened resin from a corner of a resin-impregnated archaeological sediment slab. Axlor Middle Palaeolithic site, N Spain, thin section AX-18-1d
CS	DD, LS	Burnt bone-rich facies from Layer XXIV at Crvena Stijena Middle Palaeolithic site, Western Montenegro, thin section CS-MM2-B4
SMS	DD	Organic-rich facies from Layer N4 E2 at Cape Espenberg, Northwestern Alaska, thin section SMS-1
AX-1 AX-2 AX-3	DD, LS	Organic-rich facies from Layer N, Axlor Middle Palaeolithic site, Northern Spain, thin section AX-18-1d
Salt-1	DD, LS	Abundant charred plant tissue from Layer XII at El Salt Middle Palaeolithic site, Eastern Spain, thin section SALT-08-21
Salt-2	DD, LS	Abundant charred plant tissue at El Salt Middle Palaeolithic site, Eastern Spain, thin section SALT-08-13
SC	DD, LS	Experimentally produced salmon skin char

*DD* drill dust from hardened resin or resin-impregnated block, *LS* loose sediment collected from the same facies, adjacent to the micromorphology block.

The archaeological samples were collected from four different sites: El Salt (Alcoy, eastern Spain), Axlor Cave (Dima, northern Spain), Crvena Stijena (Petrovići, western Montenegro) and Rising Whale site (Cape Espenberg, northwestern Alaska)^73–76^. El Salt, Axlor Cave and Crvena Stijena are Middle Paleolithic sites whilst Rising Whale site is more recent (from the late twelfth century A.D. to the early fourteenth century A.D.)^62^.

### Thin section manufacture

The thin sections were produced following the protocol in Leierer et al.^77^ using PALATAL P4-01 polyester resin mixed with styrene monomer and Methyl Ethyl Ketone Peroxide (MEKP) as catalyst. The slabs from El Salt site were sent to Spectrum Petrographics Inc. for thin section manufacture (5 × 7 × 0.0030 cm), while the ones from Cape Espenberg, Axlor and Crvena Stijena were manufactured into 6 × 9 × 0.003 cm thin sections by us at the AMBILAB facilities, University of La Laguna.

### Chemicals

Extraction, separation and analysis of the different lipid enriched fractions were performed using HPLC grade solvents including n-hexane, DCM, ethyl acetate and methanol (MeOH). BSTFA + TCMS (99:1 v/v; Sigma-Aldrich, Germany) and acetonitrile were used to derivatize the n-ketones and n-alkanols enriched fractions to their trimethylsilyl (TMS) ester homologues^78,79^. In case of fatty acids, the derivatization to its fatty acid methyl esters (FAMEs) was performed using MeOH and H_2_SO_4_^80,81^. Internal standards were used for GC–MS analysis (3 µL of 5α-androstane 400 mg/L in 150 µL of sample for F1 and F2; 1 µL of 5α-androstan-3-ol 400 mg/L in 50 µL of sample for F3 and F4 and 1 µL of methyl-C_19:0_ 400 mg/L in 50 µL of sample for F5 and F6) and also injections of several types of analytical standards (see more information in Sup-4) were performed to compare its signals with those obtained in the samples analyzed.

### Lipid extraction, separation and analysis

Lipid extraction and GC–MS analysis were carried out adapting the methodology described in Leierer et al.^82^. We carried out lipid biomarker analysis on DD and LS samples from our sample set, briefly explained below.

**Figure 4:**
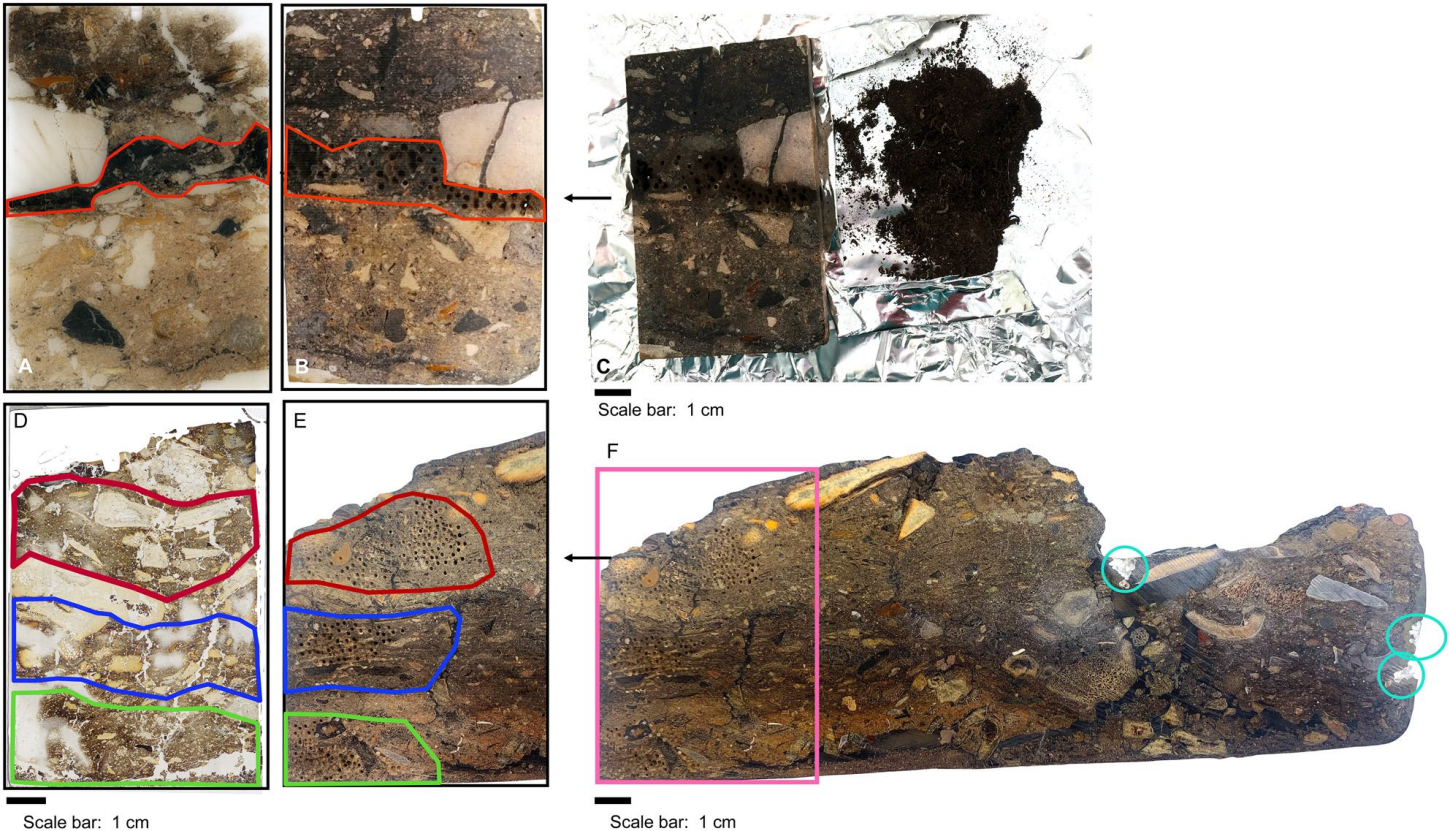
Example of drill dust collected from Salt-10-13 and Axlor slabs: (**A**) Flatbed scan image of a thin section from Salt-10-13 with area of interest outlined in red. (**B**) 1 cm-thick resin-impregnated slab of Salt-10-13, which is the mirror image of (**A**) and (**C**) Drill dust (0.2 g) obtained from the drilling process. (**D**) Flatbed scan image of thin section from Axlor-18–1 with areas of interest outlined in red, blue and green. (**E**) 1 cm-thick resin-impregnated slab of Axlor-18-1 with drill marks and (**C**) Axlor-18-1 block with the drilling area outlined in pink and resin control areas circled in blue.

Prior to extraction, the loose sediment samples (about 5.5 g) were dried at 60 °C for 48 h in a heating oven (Nabertherm GmbH, Lilienthal, Germany) and homogenized with an agate mortar. The resin-impregnated slabs were locally cleaned with DCM to eliminate surface impurities and micro-drilled in areas with high organic content according to prior micromorphological observation (Fig. 4). A Proxxon Micromote 50/E (Proxxon, Wecker, Germany) was used to obtain 0.2 g of drill dust for GC–MS analysis. We used a 1.0 mm multi-layered electroplated diamond solid thin drill tip (UKAM, Valencia, CA-USA) at minimum speed (5000 rpm/min).

We extracted around 0.2 g of DD samples and proximately 5 g for LS samples, using a mixture of DCM:MeOH (9:1 v/v; 10 mL for DD samples and 50 mL for LS samples) to obtain the TLE. TLE was fractionated through a silica column using several mobile phases to obtain different lipid enriched fractions (Table 4). For the resin control samples, we disregarded the fractions eluting with a high percentage of DCM (F3 and F4) because we observed serious obstructions in the silica chromatography column, possibly related with a change in the aggregation state of the resin drill dust.

All the fractions were analyzed twice at the Archaeological Micromorphology and Biomarker Research Laboratory (AMBILAB, Universidad de La Laguna, Spain) using a GC–MS coupled to a single quadrupole MS with an electron impact interface (Agilent 7890B-5977A MSD) equipped with a multimode injector (Agilent Technologies, Waldbronn, Germany). To separate the analytes, an HP-5MS capillary column (30 m length × 0.25 mm i.d., 0.25-μm film thickness) was employed (see Sup-1). We used MassHunter Workstation Software (Agilent Technologies, Waldbronn, Germany) to control the GC–MS and for data acquisition. Archaeological lipid biomarkers and resin constituents were identified according to their polarity, retention times and NIST Mass Spectra Database (v.14) matches. Compounds with less than 30% probability were disregarded, those with more than 50% probability and high values of match and R match (above 800) were included, and those from 30 to 50% probability with high values of match and R match were examined with additional information based on specialized literature.

### Stable isotope δ^13^C analysis

We performed Specific Isotope Analysis (CSIA), a widely used technique in archaeological science^21–23^, to determine the δ^13^C values of C_16:0_ and C_18:0_ fatty acids in our sample set. We followed the methodology applied by Jambrina-Enríquez et al.^22^. The samples were analyzed at the Archaeological Micromorphology and Biomarker Research Laboratory (AMBILAB, Universidad de La Laguna, Spain) using a GC-IRMS setup consisting of a Thermo Scientific Isotope Ratio Mass Spectrometer Delta V Advantage (Thermo Scientific, Walthman, MA, USA) coupled to a GC Trace 1310 through a Conflo IV interface with a temperature converter GC Isolink II. The chromatograph was equipped with a Trace Gold 5-MS capillary column (30 m, 0.25 mm i.d., 0.25 µm phase thickness, Thermo Scientific) and He was used as carrier gas (see Sup-1). Every sample was injected three times and a FAMEs standard mixture comprising from C_14:0_ methyl ester to C_20:0_ ethyl ester of known isotopic value was injected prior each set of analysis to ensure that either, the combustion furnace or instruments were running correctly. The admitted standard deviation of the FAMEs standard mixture was 0.5 as maximum for the analysis. Corrections of the isotopic values of the fatty acids were performed in all the samples to avoid biases derived from the isotopic signal of the methyl groups. This correction was made according the equation established by Goodman and Brenna^83^. Considering that the fatty acids used in resin mixtures come from modern plant oils, we also corrected δ^13^C values in all the resin related samples to assess the decrease in atmospheric ^13^CO_2_, or Suess effect^22,84^. The correction factor applied was + 1.9‰ to match the archaeological values, considering a preindustrial atmosphere with δ^13^C − 6.4‰^85^ and δ^13^C − 8.5‰ as the value at the time of sampling^86^. Isotopic values are expressed in the common notation in ‰ relative to the Vienna Pee Dee Belemnite (VPDB).

### Statistical analysis

Statistical analysis of the resulting data was carried out to evaluate the influence of the resin on the n-alkane profiles of the AX1, AX2, AX3 and CS DD samples, as well as on the fatty acid δ^13^C_16:0_ and δ^13^C_18:0_ isotopic values. Normality in the distribution of the data was checked through normality tests. As the values did not follow a normal distribution, we used XLSTAT v. 2020.2.2 software to apply two types of non-parametrical tests: Mann Whitney and comparison between related samples using a bilateral test and the Wilcoxon Index, both at 95% confidence. To assess if the difference between the LS and DD samples affects the discrimination between different possible sources of fatty acids, we used Statistica software v.15 to represent two-dimensional (δ^13^C_16:0_ *vs* δ^13^C_18:0_) 95% confidence ellipse plots.

## Supplementary information

Supplementary Information.
