# Supplementary material for: Micro-contextual identification of archaeological lipid biomarkers using resin-impregnated sediment slabs
Source: Sci Rep. 2020 Nov 25;10:20574. doi: 10.1038/s41598-020-77257-x (PMC7689525; doi:10.1038/s41598-020-77257-x)
Supplement: Supplementary file 1 — Supplementary Information. [file 41598_2020_77257_MOESM1_ESM.docx]

**Micro-contextual identification of archaeological lipid biomarkers using resin-impregnated sediment slabs**

Caterina Rodríguez de Vera^1*^, Antonio V. Herrera-Herrera^1^, Margarita Jambrina-Enríquez^1,2^, Santiago Sossa-Ríos^3,4^, Jesús González-Urquijo^5^, Talia Lazuen^6^, Marine Vanlandeghem^7^, Claire Alix^8^, Gilliane Monnier^9^, Goran Pajović^10^, Gilbert Tostevin^9^, Carolina Mallol^1,11^

1. Archaeological Micromorphology and Biomarkers Laboratory (AMBI Lab), Instituto Universitario de Bio-Orgánica “Antonio González”, Universidad de La Laguna, Tenerife, Spain.
2. Departamento de Biología Animal, Edafología y Geología, Universidad de La Laguna, Tenerife, Spain.
3. Universitat Rovira i Virgili, Departamento de Historia e Historia del Arte, Avenida de Cataluña, 35, 43002, Tarragona, Spain.
4. Institut Català de Paleoecología Humana i Evolució Social (IPHES), Zona Educacional 4, Campus Sescelades Universitat Rovira i Virgili (Edificio W3), 43007, Tarragona, Spain.
5. Instituto Internacional de Investigaciones Prehistóricas de Cantabria, IIIPC-University of Cantabria, Edificio Interfacultativo, Universidad de Cantabria, Avenida de Los Castros, 52, 39005, Santander, Spain.
6. Université de Bordeaux, CNRS, MCC, PACEA, UMR 5199, 33600 Pessac Cedex, France.
7. Université Paris 1 Panthéon Sorbonne, UMR 7041 ArScAn, 21 allée de l’université, 92023, Nanterre Cedex, France.
8. Université Paris 1 Panthéon Sorbonne, 8096 ArchAm, 21 allée de l’université, 92023, Nanterre Cedex, France.
9. Department of Anthropology, University of Minnesota, Minneapolis, MN, USA.
10. National Museum of Montenegro, Novice Cerovića, 7, 81250, Cetinje, Montenegro.
11. Departamento de Geografía e Historia, UDI Prehistoria, Arqueología e Historia Antigua, Facultad de Geografía e Historia, Universidad de La Laguna, Tenerife, Spain.

*corresponding author: crodrive@ull.edu.es

**Sup-1**: GC-MS and GC-IRMS separation methods.

- - 1. *GC-MS separation method*

To separate the analytes, GC oven was set initially at 70°C during 2 min, then was heated with a heating rate of 12°C/min up to 140°C and finally was heated up to 320°C with a heating rate of 3°C/min and held for 15 min. One microlitre of sample was injected in split mode (ratio 5:1) and the multimode injector was held at 70°C for 0.85 min and then heated up to 300°C at a heating rate of 720°C/min.

- - 1. *Stable isotope* δ*^13^C separation method*

The temperature program used started with 2 min at 70ºC before an increase until 140 ºC (heating rate, h. r., 12 ºC/min) and held to this temperature during 2 min. In the last step, an increase until 320 ºC was programmed with a h. r. of 3 ºC/min and held for 15 min. The temperature in the combustion reactor was maintained at 1000 ºC. The injection of the samples was made in splitless mode through a Programmed Temperature Vaporising (PTV) injector with an evaporation step from 60 ºC to 79 ºC (h. r. 10 ºC/min and held 30s), before a transfer stage at 325 ºC/ min (h. r. 10ºC/s) and a final cleaning stage at 350 ºC (h. r. 14 ºC/s, held 3 min). The volume of injection was 1µL of each sample.

**Sup-2**: Resin constituents identified in the resin-related samples.

| Compound | RT (min) | Key ion (*m/z*) | Compound formula | NIST number | CAS | Molecular weight | Probability (%) | Use | Detected in Fraction | | | | | |
| --- | --- | --- | --- | --- | --- | --- | --- | --- | --- | --- | --- | --- | --- | --- |
|  |  |  |  |  |  |  |  |  | 1 | 2 | 3 | 4 | 5 | 6 |
| Benzoic Acid | 6,5 | 105, 179 | C_7_H_6_O_2_ | 290514 | 65-85-0 | 122 | 34,8 | Basic constituent ^[1]^ |  | x | x | x | x |  |
| Hydrocinnamic acid* | 7 | 73 | C_9_H_10_O_2_ | 333897 | 501-52-0 | 150 | 45,0 | Antioxidant ^[2]^ |  |  |  | x |  |  |
| Phthalic anhydride | 7,2 | 76 | C_8_H_4_O_3_ | 160694 | 85-44-9 | 148 | 56,4 | Basic constituent ^[3,4]^ |  | x | x | x | x | x |
| Phthalic acid | 7,2 | 104 | C_8_H_6_O_4_ | 78990 | 88-99-3 | 166 | 54,9 | Plasticizer ^[3]^ |  | x | x | x | x | x |
| 1-Phenyl-1,2-ethanediol (styrene glycol) | 8,6 | 107, 179 | C_8_H_10_O_2_ | 229983 | 93-56-1 | 138 | 82,6 | Antiplasticizer ^[5]^ |  |  |  | x |  |  |
| Dimethyl phthalate | 8,9 | 163 | C_10_H_10_O_4_ | 378702 | 131-11-3 | 194 | 64,9 | Plasticizer ^[4,6,7]^ |  |  | x | x | x | x |
| 3-Nitrophthalhydrazide | 9,5 | 207, 104 | C_8_H_5_N_3_O_4_ | 238753 | 3682-15-3 | 207 | 54,2 | Curing agent ^[8]^ |  |  |  | x | x |  |
| Phenylacetic acid | 10,4 | 73 | C_8_H_8_O_2_ | 149702 | 2078-18-4 | 208 | 91,3 | Byproduct ^[9]^ |  |  |  | x |  |  |
| Monomethyl phthalate | 10,7 | 89 | C_9_H_8_O_4_ | 135497 | 4376-18-5 | 180 | 94,7 | Byproduct ^[10]^ |  |  |  | x | x | x |
| 2-Butenedioic acid (Fumaric acid) | 11,6 | 73, 98 | C_4_H_4_O_4_ | 194179 | 110-17-8 | 116 | 40,5 | Fortifier ^[1,4]^ |  |  |  | x |  | x |
| N-aminophthalimide | 12,1 | 162 | C_8_H_6_N_2_O_2_ | 231769 | 1875-48-5 | 162 | 41,3 | Detected in plastics ^[11]^ |  |  |  |  | x |  |
| Phthalhydrazide | 12,2 | 162 | C_8_H_6_N_2_O_2_ | 229780 | 1445-69-8 | 162 | 59,6 | Curing agent ^[12,13]^ |  |  |  |  | x |  |
| Methyl 2-ethylhexyl phthalate | 13,5 | 163 | C_17_H_24_O_4_ | 373833 | 56166-83-7 | 292 | 30,6 | Plasticizer ^[10,14,15]^ |  |  |  |  | x | x |
| Diallyl phthalate | 13,9 | 41 | C_14_H_14_O_4_ | 228069 | 131-17-9 | 246 | 74,2 | Plasticizer ^[4]^ |  |  |  | x |  |  |
| 1,4-pentanedione 1-phenyl | 15,1 | 105 | C_11_H_12_O_2_ | 342743 | 583-05-1 | 176 | 46,0 | Co-polymer ^[16,17]^ |  |  |  | x |  | x |
| Azelaic acid* | 15,5 | 73 | C_9_H_16_O_4_ | 352564 | 123-99-9 | 188 | 77,0 | Basic constituent ^[1]^ |  |  |  | x | x |  |
| 2-((2-(2-Methoxyethoxy)ethoxy)carbonyl)benzoic acid | 16,8 | 149 | C_13_H_16_O_6_ | 373521 | 207790-01-0 | 268 | 46,5 | Resin constituent ^[18]^ |  |  |  | x |  |  |
| Propanoic acid, 2-methyl-, 2,2-dimethyl-1-(1-methylethyl)-1,3-propanediyl ester (Kodaflex txib) | 16,9 | 71 | C_16_H_30_O_4_ | 417295 | 6846-50-0 | 286 | 92,2 | Plasticizer ^[19]^ |  |  |  | x |  |  |
| Monoethyl phthalate | 18,2 | 75 | C_10_H_10_O_4_ | 373530 | 55530-57-9 | 266 | 91,7 | Hydrolyzed plasticizer ^[10,14]^ |  |  |  | x |  |  |
| Monoisopropyl phthalate | 19,1 | 223 | C_11_H_12_O_4_ | 373524 | 35118-50-4 | 208 | 60,0 | Plasticizer ^[20]^ |  |  |  | x |  |  |
| Monoallyl phthalate | 20,4 | 41, 149 | C_11_H_10_O_4_ | 373676 | 388-14-2 | 206 | 98,6 | Byproduct ^[21]^ |  |  |  | x |  |  |
| Trans-1,2-diphenylcyclobutane | 24,1 | 104 | [C_16_H_16_](https://pubchem.ncbi.nlm.nih.gov/#query=C16H16) | 62824 | 20071-09-4 | 208 | 57,6 | Degradation product ^[22]^ |  | x |  | x | x |  |
| Chalcone | 28,2 | 207 | C_15_H_12_O | 22584 | 94-41-7 | 208 | 65,4 | UV stabilizer ^[23]^ |  |  |  |  |  | x |
| 1,3-Diphenyl-2-buten-1-one (β-Methylchalcone) | 28,3 | 221 | C_16_H_14_O | 135187 | 54435-79-9 | 222 | 32,7 | Plasticizer ^[24]^ |  |  |  | x |  |  |
| Benzene, 1,1',1''-[5-methyl-1-pentene-1,3,5-triyl]tris- | 33 | 106 | C_24_H_24_ | 402265 | 61909-63-5 | 312 | 73,2 | Detected in polystyrene ^[9]^ |  |  |  |  |  |  |
| α N-normethadol | 33,7 | 91 | C_20_H_27_NO | 248097 | 38455-85-5 | 297 | 45,4 | Degradation product ^[9]^ |  | x |  |  |  |  |
| 1-Propene, 3-(2-cyclopentenyl)-2-methyl-1,1-diphenyl- | 33,8 | 91, 207 | C_21_H_22_ | 154233 |  | 274 | 33,2 | Detected in plastics ^[9,11]^ |  | x | x |  | x |  |
| Thiocarbamic acid, N,N-dimethyl, S-1,3-diphenyl-2-butenyl ester | 34,3 | 91, 207 | C_19_H_21_NOS | 192892 |  | 311 | 38,6 | Detected in plastics ^[9]^ |  | x |  |  | x |  |
| Diisooctyl phthalate | 34,6 | 149 | C_24_H_38_O_4_ | 62126 | 27554-26-3 | 390 | 46,3 | Plasticizer ^[25]^ | x | x | x |  | x |  |
| Triphenyl stibine | 39,3 | 198 | [C_18_H_15_Sb](https://pubchem.ncbi.nlm.nih.gov/#query=C18H15Sb) | 245770 | 603-36-1 | 353 | 92,5 | Flame retardant ^[12,26]^ |  | x | x |  | x |  |
| Hexanedioic acid, bis(2-ethylhexyl) ester (Kodaflex DOA) | 39,7 | 129 | C_22_H_42_O_4_ | 291314 | 103-23-1 | 370 | 72,9 | Plasticizer ^[27]^ |  | x |  |  |  |  |
| Allyl methyl phthalate | 43,7 | 163 | C_12_H_12_O_4_ | 373891 |  | 220 | 87,6 | Plasticizer ^[28]^ |  |  |  | x |  |  |
| Methyl phthalyl ethyl glycolate | 43,9 | 163 | C_13_H_14_O_6_ | 232709 | 85-71-2 | 266 | 36,3 | Plasticizer ^[29]^ |  |  |  |  | x |  |
| 2-(2'-Hydroxy-3',5'-di-tert-amylphenyl)benzotriazole | 46,1 | 322 | C_22_H_29_N_3_O | 404119 | 25973-55-1 | 351 | 98,0 | UV stabilizer ^[17]^ |  |  |  |  | x | x |
| Benzophenone-3 | 20,4 | 227 | C_14_H_12_O_3_ | 291141 | 131-57-7 | 228 | 81,8 | UV stabilizer ^[30]^ |  |  |  | x |  |  |
| Cinnamic acid | 9 | 131 | C_9_H_8_O_2_ | 5883 | 140-10-3 | 148 | 45 | UV stabilizer ^[2]^ |  |  |  |  | x |  |
| Terephthalic acid | 11,1 | 163 | C_8_H_6_O_4_ | 290670 | 100-21-0 | 166 | 72 | Plasticizer ^[3]^ |  |  |  |  | x |  |
| Isophthalic acid | 11,2 | 163 | C_8_H_6_O_4_ | 107396 | 121-91-5 | 166 | 52 | Basic constituent ^[1,3,31]^ |  |  |  |  | x |  |
| 2-naphthoic acid | 18,1 | 128 | C_11_H_8_O_2_ | 68123 | 93-09-4 | 172 | 43 | Resin constituent ^[18]^ |  |  |  |  | x |  |
| Benzenepropanoic acid, 3,5, bis (1,1-dimethylethyl), 4-hydroxy-, octadecyl ester | 60,4 | 57 | C_35_H_62_O_3_ | 384330 | 2082-79-3 | 530 | 97 | Antioxidant ^[32]^ |  |  |  |  | x |  |
| 2,4-di-tert-butylphenol | 11,2 | 191 | C_14_H_22_O | 133233 | 96-76-4 | 206 | 64,3 | Antioxidant ^[17]^ |  |  |  |  | x |  |

Compounds with (*) were detected as its derivative homologous.

**Sup-3**: Lipid biomarkers identified in the present study.

| Sample |  |  | Fraction |  |  |
| --- | --- | --- | --- | --- | --- |
|  | F1  (n-Alkanes) | F2  (Aromatic compounds) | F3  (n-Ketones) | F4  (Alcohols) | F5 & F6  (Fatty acids and other more polar compounds) |
| AX1 LS | C_21_-C_31_ | - | - | nC_22_-OH -nC_30_-OH (even-numbered carbon chain length)  1-Monostearin  1-Monopalmitin  Glycerol | C_16:0_, C_18:0_ |
| AX1 DD | C_21_-C_31_ | - | - | Glycerol | C_16:0_, C_18:0_ |
| AX2 LS | C_21_-C_33_ | - | - | nC_22_-OH -nC_30_-OH (even-numbered carbon chain length)  1-Monostearin  1-Monopalmitin  Glycerol  β -sitosterol | C_16:0_, C_18:0_ |
| AX2 DD | C_21_-C_33_ | - | - | nC_20_-OH nC_26_-OH  1-Monopalmitin  Glycerol | C_16:0_, C_18:0_ |
| AX3 LS | C_21_-C_33_ | - | - | nC_22_-OH -nC_30_-OH (even-numbered carbon chain length) | C_16:0_, C_18:0_ |
| AX3 DD | C_21_-C_33_ | - | - | nC_20_-OH, nC_26_-OH, nC_28_-OH  1-Monopalmitin  Glycerol | C_16:0_, C_18:0_ |
| CS LS | C_19_-C_33_ | - | - | nC_16_-OH -nC_30_-OH (even-numbered carbon chain length)  1-Monopalmitin  Glycerol monostearate | C_14:0_, C_16:0_, C_18:0_ |
| CS DD | C_19_-C_33_ | - | - | nC_20_-OH, nC_30_-OH | C_14:0_, C_16:0_, C_18:0_ |
| SC | - | - | - |  | C_14:0_, C_16:0_, C_16:1Δ9_, C_18:0_, C_18:1Δ9_, C_18:1Δ11_, C_18:2Δ9,11,_ C_20:0,_ C_20:4Δ5,8,11,14*,_ nonanedioic acid,  undecanedioic acid |
| SCDD | - | Pyrene | - | Cholesterol | C_14:0_, C_16:0_, C_16:1Δ9_, C_18:0_, C_18:1Δ9_, C_18:1Δ11_, C_18:2Δ9,11,_ C_20:0,_ C_15:0_, C_17:0_, C_18:2__Δ9,12_, C_20:1Δ11_, C_22:0_, decanedioic acid, undecanedioic acid |
| SMS DD | - | - | 16-Hentriacontanone  18- Pentatriacontanone | nC_14_-OH, nC_16_-OH, nC_18_-OH | C_14:0_-C_24:0_ (even numbered carbon chain length)  10-hidroxy-C_16:0_  10-hidroxy-C_18:0_ |
| Salt-1 LS | C_18_-C_33_ | - | - | nC_14_-OH, nC_16_-OH, nC_18_-OH | C_16:0_, C_18:0_ |
| Salt-1 DD | C_18_-C_29_ | - | - | - | C_16:0_, C_18:0_ and oleanolic acid |
| Salt-2 LS | - | - | - | - | C_16:0_, C_18:0_ |
| Salt-2 DD | - | - | - | - | C_16:0_, C_18:0_ |
| R1 | C_22_-C_33_ | - | - | - | C_16:0_, C_18:0_ |
| R2 | - | - | - | - | C_16:0_, C_18:0_ |
| R3 | - | - | - | - | C_16:0_, C_18:0_ |
| R4 | - | - | - | - | C_16:0_, C_18:0_ |

*The delta symbol (Δ) indicates the position of the unsaturation.

**Sup-4**: List of standards used in the present study.

| n-alkanes (mix C_4_-C_40_ standard, solution) | | | | | |
| --- | --- | --- | --- | --- | --- |
| **Analite** | **Compound formula** | **CAS Number** | **Purity (%)** | **Supplier** | **Concentration (mg/L)** |
| n-Octane | C_8_H_18_ | 111-65-9 | 99,4 | Supelco | 500,0 |
| n-Nonane | C_9_H_20_ | 111-84-2 | 99,9 | Supelco | 500,0 |
| n-Decane | C_10_H_22_ | 124-18-5 | 99,9 | Supelco | 500,4 |
| n-Undecane | C_11_H_24_ | 1120-21-4 | 99,6 | Supelco | 500,0 |
| n-Dodecane | C_12_H_26_ | 112-40-3 | 99,6 | Supelco | 500,0 |
| n-Tridecane | C_13_H_28_ | 629-50-5 | 99,9 | Supelco | 500,0 |
| n-Tetradecane | C_14_H_30_ | 629-59-4 | 99,5 | Supelco | 500,4 |
| n-Pentadecane | C_15_H_32_ | 629-62-9 | 99,6 | Supelco | 500,0 |
| n-Hexadecane | C_16_H_34_ | 544-79-3 | 99,9 | Supelco | 500,0 |
| n-Heptadecane | C_17_H_36_ | 629-78-7 | 99,8 | Supelco | 500,0 |
| Pristane | C_19_H_40_ | 1921-70-6 | 99,6 | Supelco | 500,4 |
| n-Octadecane | C_18_H_38_ | 593-45-3 | 99,4 | Supelco | 500,4 |
| Phytane | C_20_H_42_ | 638-36-8 | 96,4 | Supelco | 500,1 |
| n-Nonadecane | C_19_H_40_ | 629-92-5 | 99,7 | Supelco | 500,4 |
| n-Eicosane | C_20_H_42_ | 112-95-8 | 99,2 | Supelco | 500,4 |
| n-Heneicosane | C_21_H_44_ | 629-94-7 | 99,9 | Supelco | 500,4 |
| n-Docosane | C_22_H_46_ | 629-97-0 | 99,9 | Supelco | 500,0 |
| n-Tricosane | C_23_H_48_ | 638-67-5 | 99,9 | Supelco | 500,4 |
| n-Tetracosane | C_24_H_50_ | 646-31-1 | 99,4 | Supelco | 500,0 |
| n-Pentacosane | C_25_H_52_ | 629-99-2 | 99,3 | Supelco | 500,4 |
| n-Hexacosane | C_26_H_54_ | 630-01-3 | 99,6 | Supelco | 500,0 |
| n-Heptacosane | C_27_H_56_ | 593-49-7 | 99,6 | Supelco | 500,4 |
| n-Octacosane | C_28_H_58_ | 630-02-4 | 99,9 | Supelco | 500,0 |
| n-Nonacosane | C_29_H_60_ | 630-03-5 | 99,7 | Supelco | 500,4 |
| n-Triacontane | C_30_H_62_ | 638-68-6 | 98,7 | Supelco | 500,8 |
| n-Hentriacontane | C_31_H_64_ | 630-04-6 | 98,5 | Supelco | 500,8 |
| n-Dotriacontane | C_32_H_66_ | 544-85-4 | 98,9 | Supelco | 500,4 |
| n-Tritriacontane | C_33_H_68_ | 630-05-7 | 97,8 | Supelco | 500,0 |
| n-Tetratriacontane | C_34_H_70_ | 14167-59-0 | 99,9 | Supelco | 500,0 |
| n-Pentatriacontane | C_35_H_72_ | 630-07-9 | 98,3 | Supelco | 500,8 |
| n-Hexatriacontane | C_36_H_74_ | 630-06-8 | 99,9 | Supelco | 500,0 |
| Heptatriacontane | C_37_H_76_ | 7194-84-5 | 99,9 | Supelco | 500,0 |
| Octatriacontane | C_38_H_78_ | 7194-85-6 | 97,1 | Supelco | 500,2 |
| Nonatriacontane | C_39_H_80_ | 7194-86-7 | 90,1 | Supelco | 500,2 |
| n-tetracontane | C_40_H_82_ | 4181-95-7 | 99,9 | Supelco | 500,0 |
| Aromatics (individual standards, powder) | | | | | |
| **Analite** | **Compound formula** | **CAS Number** | **Purity (%)** | **Supplier** | **Mass (g)** |
| Naphthalene | C_10_H_8_ | 91-20-3 | - | Sigma Aldrich | 0.25 |
| Antrhacene | C_14_H_10_ | 120-12-7 | CRM* | Sigma Aldrich | 0.10 |
| Phrenanthrene | C_14_H_10_ | 85-01-8 | 99 | Pr Ehaenstata | 0.05 |
| Ketones (individual standard, powder) | | | | | |
| **Analite** | **Compound formula** | **CAS Number** | **Purity (%)** | **Supplier** | **Mass (g)** |
| 14-Nonacosanone | C_29_H_58_O | 34394-11-1 | - | Syntester Research Group | 2 |
| 16-Hentriacontanone | C_31_H_62_O | 502-73-8 | 95 | Tokyo Chemical Industry | 25 |
| 16-Tritriacontanone | C_33_H_66_O | 15740-35-9 | - | Syntester Research Group | 2 |
| 18-Pentatriacontanone | C_35_H_70_O | 504-53-0 | 85 | Alfa Aesar | 25 |
| Alcohols (individual standards, powder) | | | | | |
| **Analite** | **Compound formula** | **CAS Number** | **Purity (%)** | **Supplier** | **Mass (g)** |
| 1-Tetradecanol | C_14_HO | 112-72-1 | 99 | Sigma Aldrich | 1 |
| 1-Hexadecanol | C_16_HO | 36653-82-4 | 99.5 | PR Ehrenstrofer | 0.25 |
| Cholesterol | C_27_H_46_O | 57-88-5 | 99 | Sigma Aldrich | 0.5 |
| ß-sitosterol | C_29_H_50_O | 83-46-5 | 98.3 | Supelco | 100* |
| 3-coprostanol | C_27_H_48_O | 360-68-9 | 98 | Sigma Aldrich | 0.1 |
| Stigmastanol | C_29_H_52_O | 83-48-7 | 95 | Cayman Chemical Company | 1 |
| FAMES (mix C_4:0_-C_24:0_ standard, solution) | | | | | |
| **Analite** | **Compound formula** | **CAS Number** | **Purity (%)** | **Supplier** | **Concentration (mg/L)** |
| Methyl butanoate | C_5_H_10_O_2_ | 623-42-7 | 99,9 | Supelco | 399,6 |
| Methyl hexanoate | C_7_H_14_O_2_ | 106-70-7 | 99,9 | Supelco | 399,6 |
| Methyl octanoate | C_9_H_18_O_2_ | 111-11-5 | 99.9 | Supelco | 399,6 |
| Methyl decanoate | C_11_H_22_O_2_ | 110-42-9 | 99,9 | Supelco | 399,6 |
| Methyl undecanoate | C_12_H_24_O_2_ | 1731-86-8 | 99,9 | Supelco | 199,8 |
| Methyl dodecanoate | C_13_H_26_O_2_ | 111-82-0 | 99,9 | Supelco | 399,6 |
| Methyl tridecanoate | C_14_H_28_O_2_ | 1731-88-0 | 98,8 | Supelco | 197,7 |
| Methyl tetradecanoate | C_15_H_30_O_2_ | 124-10-7 | 99,9 | Supelco | 399,6 |
| Methyl *cis*-9-tetradecenoate | C_15_H_28_O_2_ | 56219-06-8 | 99,6 | Supelco | 199,2 |
| Methyl pentadecanoate | C_16_H_33_O_2_ | 7132-64-1 | 99,9 | Supelco | 199,8 |
| Methyl *cis*-10- pentadecenoate | C_16_H_30_O_2_ | 90176-52-6 | 99,0 | Supelco | 198,0 |
| Methyl hexadecanoate | C_17_H_34_O_2_ | 112-39-0 | 99,9 | Supelco | 599,5 |
| Methyl *cis*-9- hexadecenoate | C_17_H_32_O_2_ | 1120-25-8 | 99,9 | Supelco | 199,9 |
| Methyl heptadecanoate | C_18_H_36_O_2_ | 1731-92-6 | 99,6 | Supelco | 199,2 |
| Methyl *cis*-10-heptadecenoate | C_18_H_34_O_2_ | 75190-82-8 | 99,9 | Supelco | 199,8 |
| Methyl octadecanoate | C_19_H_38_O_2_ | 112-61-8 | 99.9 | Supelco | 399,6 |
| Mehtyl *trans-*9-elaidate | C_19_H_36_O_2_ | 1937-62-8 | 99,9 | Supelco | 199,8 |
| Methyl c*is*-9-octadecenoate | C_19_H_36_O_2_ | 112-62-9 | 99,9 | Supelco | 399,6 |
| Methyl t*rans, trans*-9,12- octadecadienoate | C_19_H_34_O_2_ | 2566-97-4 | 99,9 | Supelco | 199,8 |
| Methyl *cis, cis*-9,12- octadecadienoate | C_19_H_34_O_2_ | 112-63-0 | 98,9 | Supelco | 197,9 |
| Methyl c*is, cis, cis*-9,12-15- octadecatrienoate | C_19_H_32_O_2_ | 301-00-8 | 99,9 | Supelco | 199,8 |
| Methyl γ-*cis, cis, cis*-9,12,15- octadecatrienoate | C_19_H_32_O_2_ | 16326-32-2 | 98,8 | Supelco | 197,6 |
| Methyl all *cis-*5,8,11,14,17-eicosapentaenoate | C_21_H_32_O_2_ | 2734-47-6 | 99,9 | Supelco | 199,8 |
| Methyl all *cis-*5,8,11,14-eicosatetraenoate | C_21_H_34_O_2_ | 2566-89-4 | 99,9 | Supelco | 199,8 |
| Methyl *cis, cis, cis-*8,11,14- eicosatrienoate | C_21_H_36_O_2_ | 21061-10-9 | 99,9 | Supelco | 199,8 |
| Methyl *cis, cis, cis-*11,14,17- eicosatrienoate | C_21_H_36_O_2_ | 55682-88-7 | 96,8 | Supelco | 200,0 |
| Methyl *cis, cis-*11,14- eicosadienoate | C_21_H_38_O_2_ | 2463-02-7 | 99,9 | Supelco | 199,8 |
| Methyl *cis-*11-eicosenoate | C_21_H_40_O_2_ | 2390-09-2 | 99,6 | Supelco | 199,2 |
| Methyl eicosanoate | C_21_H_42_O_2_ | 301-00-8 | 99,9 | Supelco | 199,8 |
| Methyl heneicosanoate | C_22_H_44_O_2_ | 6064-90-0 | 99,4 | Supelco | 198,8 |
| Methyl all *cis-*4-7-10-13-16-19-methyl-docosahexaenoate | C_23_H_34_O_2_ | 2566-90-7 | 99,9 | Supelco | 199,8 |
| Methyl *cis, cis-*13-16-docosadienoate | C_23_H_42_O_2_ | 61012-47-3 | 99,9 | Supelco | 199,8 |
| Methyl *cis-*13- docosenoate | C_23_H_44_O_2_ | 1120-34-9 | 99,9 | Supelco | 199,8 |
| Methyl docosanoate | C_23_H_46_O_2_ | 929-77-1 | 99,7 | Supelco | 398,8 |
| Methyl tricosanoate | C_24_H_48_O_2_ | 2433-97-8 | 99,6 | Supelco | 199,2 |
| Methyl *cis-*15-nervonate | C_25_H_48_O_2_ | 2733-88-2 | 98,4 | Supelco | 196,9 |
| Methyl tetracosanoate | C_25_H_50_O_2_ | 2442-49-1 | 99,9 | Supelco | 399,6 |

**Supplementary References**

1. G.G., E. & M.H., S. Identification of Carboxylic Acids in Alkyd and Polyester Coating Resins by Programmed Temperature Gas Chromatography. *Anal. Chem.* **34**, 1048–1052 (1962).

2. Fonseca, A. C. *et al.* Cinnamic acid derivatives as promising building blocks for advanced polymers: Synthesis, properties and applications. *Polym. Chem.* **10**, 1696–1723 (2019).

3. Graham, P. R. Phthalate Ester Plasticizers-Why and How They Are Used. *Environ. Health Perspect.* **3**, 3–12 (1973).

4. Kanerva, L., Tarvainen, K., Estlander, T. & Jolanki, R. Polyester Resins. in *Handbook of Occupational Dermatology* (eds. Kanerva, L., Elsner, P., Wahlberg, J. E. & Maibach, H. I.) (Springer Berlin Heidelberg, 2000). doi:https://doi.org/10.1007/978-3-662-07677-4.

5. Stipek, J. & Daoust, H. Plasticizers. in *Additives for Plastics* (eds. Cantow, H. J. et al.) 98 (Springer Science+Business Media New York, 1983). doi:10.1007/978-1-4419-8481-4.

6. Christia, C. *et al.* Occurrence of legacy and alternative plasticizers in indoor dust from various EU countries and implications for human exposure via dust ingestion and dermal absorption. *Environ. Res.* **171**, 204–212 (2019).

7. Galmán Graíño, S., Sendón, R., López Hernández, J. & Rodríguez-Bernaldo de Quirós, A. GC-MS Screening Analysis for the Identification of Potential Migrants in Plastic and Paper-Based Candy Wrapers. *Polymers (Basel).* **10**, 802 (2018).

8. Hansen, E., Nilsson, N. H., Lithner, D. & Lassen, C. Hazardous substances in plastic materials. *Hazard. Subst. Plast. Mater.* 148 (2013).

9. Rashid, M. M. & Sarker, M. Waste Polyethylene Terephthalate (PETE) And Polystyrene (PS) Into Fuel. *Int. J. Sci. Technol. Res.* **2**, 176–189 (2013).

10. Mose, T., Knudsen, L. E., Hedegaard, M. & Mortensen, G. K. Transplacental transfer of monomethyl phthalate and mono(2-ethylhexyl) phthalate in a human placenta perfusion system. *Int. J. Toxicol.* **26**, 221–229 (2007).

11. Kandare, E., Kandola, B. K., Price, D., Nazaré, S. & Horrocks, R. A. Study of the thermal decomposition of flame-retarded unsaturated polyester resins by thermogravimetric analysis and Py-GC/MS. *Polym. Degrad. Stab.* **93**, 1996–2006 (2008).

12. Hahladakis, J. N., Velis, C. A., Weber, R., Iacovidou, E. & Purnell, P. An overview of chemical additives present in plastics: Migration, release, fate and environmental impact during their use, disposal and recycling. *J. Hazard. Mater.* **344**, 179–199 (2018).

13. Wang, Y. *et al.* Crystallization of poly(lactic acid) enhanced by phthalhydrazide as nucleating agent. *Polym. Bull.* **70**, 2911–2922 (2013).

14. Meeker, J. D., Sathyanarayana, S. & Swan, S. H. Phthalates and other additives in plastics: human exposure and associated health outcomes. *Philos. Trans. R. Soc.* 2097–2113 (2009) doi:10.1098/rstb.2008.0268.

15. Rastogi, S. C. Gas chromatographic analysis of phthalate esters in plastic toys. *Chromatographia* **47**, 724–726 (1998).

16. Pfaendner, R. (Photo)oxidative Stabilization of Flame-Retarded Polymers. in *Polymer Green Flame Retardants* (eds. Papaspyrides, C. D. & Kiliaris, P.) 419–439 (Elsevier, 2014). doi:10.1016/C2010-0-66406-6.

17. Ash, M. & Ash, I. *Handbook of Preservatives*. (Synapse Information Resources, Inc., 2009).

18. Kolb, B. U., Jones, C. L., Olson, D. B., McKenzie, T. L. & Naismith, N. K. Brightness enhancing film comprising nanocomposite structure having improved crack resistance. 14 (2009).

19. Cain, W. S., De Wijk, R. A., Jalowayski, A. A., Pilla Caminha, G. & Schmidt, R. Odor and chemesthesis from brief exposures to TXIB. *Indoor Air* **15**, 445–457 (2005).

20. Lee, S. H., Lim, S. W. & Lee, K. H. Properties of potentially biodegradable copolyesters of (succinic acid–1,4‐butanediol)/(dimethyl terephthalate–1,4‐butanediol). *Polym. Int.* **48**, 861–867 (1999).

21. Rosenheimer, M. O., Mischke, R. A. & Griskey, R. G. Kinetics of the formation of monoallyl phthalate. *J. Appl. Chem.* **15**, 206–207 (1965).

22. Kwon, B. G. *et al.* Monitoring of styrene oligomers as indicators of polystyrene plastic pollution in the North-West Pacific Ocean. *Chemosphere* **180**, 500–505 (2017).

23. Lazópulos, S. Q., Svarc, F., Sagrera, G. & Dicelio, L. Absorption and photo-stability of substituted dibenzoylmethanes and chalcones as UVA filters. *Cosmetics* **5**, (2018).

24. Gale, D. C., Kleine, K., Abbate, A. J., Attladotir, S. M. & Pacetti, S. D. Compositions containing fast leaching plasticizers for improved perfomance of medical devices. (2010).

25. Yin, B. & Hakkarainen, M. Oligomeric Isosorbide Esters as Alternative Renewable Resource Plasticizers for PVC. *J. Appl. Polym. Sci.* **119**, 2400–2407 (2011).

26. Brydson, J. A. Polyester resins. in *Plastic Materials* 652–696 (Elsevier Ltd, 1989). doi:10.1016/C2013-0-04038-8.

27. IARC. IARC Monographs on the evaluation of carcinogenic risks to humans. *Some Industrial Chemicals* https://www.ncbi.nlm.nih.gov/books/NBK390864/ (2000).

28. Matsumoto, A., Aso, T., Tanaka, S. & Oiwa, M. Studies of the Polymerization of Diallyl Compounds - 19. Further Discussion of the Cyclocopolymerizations of Diallyl Phthalate With Monovinyl Monomers By the Use of the Model Compounds. *J Polym Sci Part A-1 Polym Chem* **11**, 2357–2363 (1973).

29. Hansen, N. M. L., Blomfeldt, T. O. J., Hedenqvist, M. S. & Plackett, D. V. Properties of plasticized composite films prepared from nanofibrillated cellulose and birch wood xylan. *Cellulose* **19**, 2015–2031 (2012).

30. Pastorelli, S., Sanches-Silva, A., Cruz, J. M., Simoneau, C. & Losada, P. P. Study of the migration of benzophenone from printed paperboard packages to cakes through different plastic films. *Eur. Food Res. Technol.* **227**, 1585–1590 (2008).

31. Bakar, M. & Djaider, F. Effect of plasticizers content on the mechanical properties of unsaturated polyester resin. *J. Thermoplast. Compos. Mater.* **20**, 53–64 (2007).

32. Neal-Kluever, A. P., Bailey, A. B. & Hatwell, K. R. Safety assessment for octadecyl 3-(3,5-di-tert-butyl-4-hydroxyphenyl)-propionate (CAS Reg. No. 2082-79-3) from use in food contact applications. *Food Chem. Toxicol.* **86**, 176–190 (2015).
